# Segregation of morphogenetic regulatory function of *Shox2* from its cell fate guardian role in sinoatrial node development

**DOI:** 10.1038/s42003-024-06039-2

**Published:** 2024-03-29

**Authors:** Hua Li, Qinghuang Tang, Tianfang Yang, Zhengsen Wang, Dainan Li, Linyan Wang, Liwen Li, Yaoyi Chen, Hai Huang, Yanding Zhang, YiPing Chen

**Affiliations:** 1 Key Laboratory of Stem Cell Engineering and Regenerative Medicine of Fujian Province University, Fujian Medical University, Fuzhou, Fujian Province 350122 PR China; 2 Department of Cell and Molecular Biology, Tulane University, New Orleans, LA 70118 USA; 3 Southern Center for Biomedical Research and Fujian Key Laboratory of Developmental and Neural Biology, College of Life Sciences, Fujian Normal University, Fuzhou, Fujian Province 350108 PR China; 4 Department of Oral Biology, School of Dental Medicine, University at Buffalo, Buffalo, NY 14214 USA; 5 Department of Stomatology, Chengdu Second People’s Hospital, Chengdu, Sichuan Province 610021 PR China; 6 Department of Biological Sciences, College of Arts and Sciences, University at Buffalo, Buffalo, NY 14260 USA

**Keywords:** Developmental biology, Cardiology

## Abstract

*Shox2* plays a vital role in the morphogenesis and physiological function of the sinoatrial node (SAN), the primary cardiac pacemaker, manifested by the formation of a hypoplastic SAN and failed differentiation of pacemaker cells in *Shox2* mutants. *Shox2* and *Nkx2-5* are co-expressed in the developing SAN and regulate the fate of the pacemaker cells through a Shox2-Nkx2-5 antagonistic mechanism. Here we show that simultaneous inactivation of *Nkx2-5* in the SAN of *Shox2* mutants (dKO) rescued the pacemaking cell fate but not the hypoplastic defects, indicating uncoupling of SAN cell fate determination and morphogenesis. Single-cell RNA-seq revealed that the presumptive SAN cells of *Shox2*^-/-^ mutants failed to activate pacemaking program but remained in a progenitor state preceding working myocardium, while both wildtype and dKO SAN cells displayed normal pacemaking cell fate with similar cellular state. Shox2 thus acts as a safeguard but not a determinant to ensure the pacemaking cell fate through the Shox2-Nkx2-5 antagonistic mechanism, which is segregated from its morphogenetic regulatory function in SAN development.

## Abstract (web summary)

Shox2 acts as a safeguard but not determinant for cardiac pacemaking cell fate via the Shox2-Nkx2-5 antagonistic mechanism, which is segregated from its morphogenetic regulatory role in sinoatrial node development.

## Introduction

The cardiac rhythm is controlled by the sinoatrial node (SAN), functionally known as the pacemaker^1–4^. It has been demonstrated that by embryonic day 7.5 (E7.5) in mice, mesenchymal cardiac progenitor cells (MCPC) possess pacemaking potential, but only a small portion of these cells eventually develop into cardiac conduction system including the SAN, with the majority of MCPC maturating into working myocardium^5–12^. The SAN primordium appears at E10.5 based on histological structure and becomes further mature and fully functional at E13.5^9,13^. Previous studies have revealed a complex genetic network involving many genes in the regulation of SAN development, including *Hcn4*, *Tbx3*, *Tbx5*, *Tbx18*, *Isl1*, *Smoc2*, *Bmp4*, and *Shox2*^4,14–17^. Among them, *Shox2*, *Tbx3*, *Isl1*, and *Hcn4* are specifically expressed in the entire developing SAN, with *Hcn4* being regarded as a functional marker of pacemaking capability^18,19^. *Tbx3* deficient mice develop a morphologically normal SAN with compromised function^20^. In contrast, *Tbx18* deficient mice form a hypoplastic yet functional SAN^12^. These threads of evidence implicate that the molecular mechanisms underlying SAN pacemaking cell fate determination and morphogenesis, a biological process that governs the development of tissue or organ shape by orchestrating the spatial arrangement of cells during embryonic development^21^, are not identical.

Previous studies showed that *Shox2* is indispensable for the physiological function and morphogenesis of the SAN, as *Shox2* null mice display hypoplasia of the venous pole structures and failed differentiation of SAN cells, embryonic bradycardia, and upregulation of chamber-program genes in the venous pole structures^22–24^. *Nkx2-5* was originally thought to be detrimental to SAN development, and *Shox2* was shown to regulate SAN development by preventing ectopic *Nkx2-5* activation in the SAN head domain^10,22,23,25,26^. Further studies showed that *Shox2* counterbalances *Nkx2-5* in the developing SAN junction domain, an interface between the SAN and atrium that is specialized by *Nkx2-5* expression, to regulate the cell fate decisions between pacemaking cells and working myocardial cells^27^. We have recently found that *Nkx2-5* inactivation in the SAN junction cells confers them with the property of the SAN head cells and impairs SAN function, but does not affect SAN morphogenesis^28^, further supporting the existence of morphogenic genetic programs in the SAN that are separated from pacemaking cell fate determination. However, the molecular mechanisms that distinctly control each process remain unknown.

## Results and discussion

### Maintenance of pacemaking cell fate in the developing SAN requires a minimal level of *Shox2* expression

**Fig. 1:**
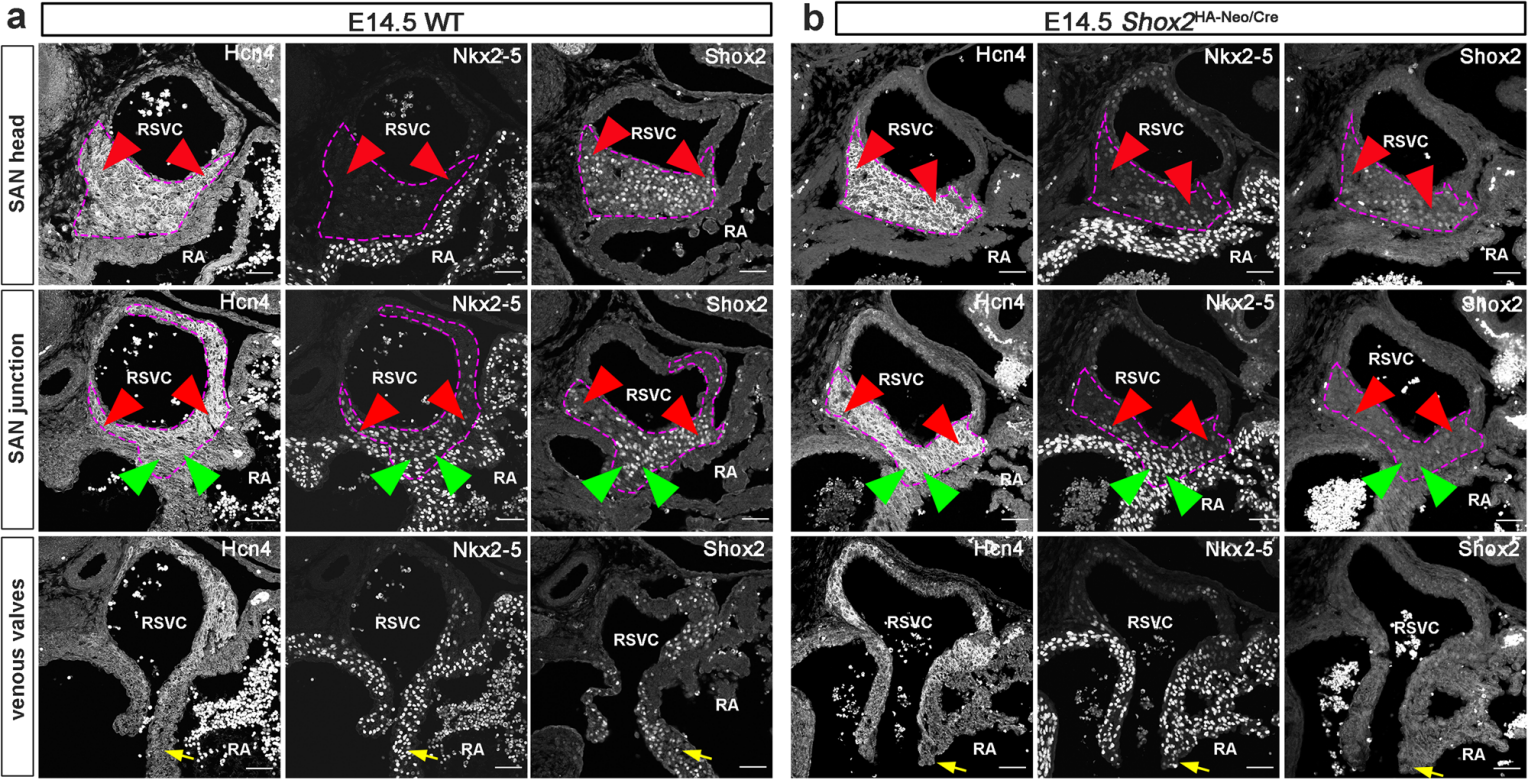
A minimal level of *Shox2* expression maintains pacemaking cell fate in the developing SAN. Immunofluorescence shows the expression of Hcn4, Nkx2-5, and Shox2 in the SAN head, SAN junction, and venous valves in E14.5 wildtype control (**a**) and *Shox2*^HA-Neo/Cre^ mice (**b**) (*n* = 3 for each genotype). Note in the whole SAN (magenta dash-lines), SAN head (red arrowheads), SAN junction (green arrowheads), and venous valves (yellow arrows), compared to controls (**a**), *Shox2*^HA-Neo/Cre^ mice (**b**) exhibited residual Shox2 expression, but visible ectopic Nkx2-5 expression in the SAN head domain and reduced size of these structures. However, Hcn4 expression was retained. RA right atrium, RSVC right superior vena cava. Bar: 50 μm.

To better understand how *Shox2* controls SAN morphogenesis and differentiation, we investigated the physiological consequence of reduced *Shox2* expression at different dosages during SAN development. We used a *Shox2-Cre* knock-in allele that express Cre recombinase instead of Shox2 as *Shox2* null allele in our studies^29^. The *Shox2-Cre* heterozygous mice appeared indistinguishable from wildtype mice, never exhibited any obvious unusual behaviors, abnormal ECG recordings, and altered gene expression profiles. In the *Shox2*^HA-Neo/+^ allele that was used as a hypomorphic allele in this study, the presence of the neomycin cassette interferes with *Shox2* expression, resulting in a reduction of *Shox2* expression to 65%^30^ (Supplementary Fig. 1a; Supplementary Data 1). A combination of different *Shox2* alleles enabled the manipulation of *Shox2* expression at different dosages (Supplementary Table 1). As Hcn4 reflects the pacemaking capability of cells in the SAN, we examined Hcn4 expression in mice carrying different compound *Shox2* alleles at various stages. We found that *Shox2*^HA-Neo/Cre^ mice, which had *Shox2* production reduced to 15% and ectopic Nkx2-5 expression (Fig. 1b) compared to wildtype mice (Fig. 1a), displayed Hcn4^+^ domains in the *Shox2*^+^ lineage derived venous pole structures (Fig. 1b). However, such mice had a reduced SAN size with retained head and junction domains and shortened venous valves (Fig. 1b). These results indicate that a 15% level of *Shox2* production compared to wildtype was sufficient for the determination and maintenance of pacemaking cell fate, but such residual dosage failed to support normal SAN morphogenesis. As compared to its requirement for normal SAN morphogenesis, the maintenance of pacemaking cell fate requires a lower dose of *Shox2*. The hypoplastic SAN morphology but with the retaining of the pacemaking cell fate in the *Shox2*^HA-Neo/Cre^ suggests the existence of distinct *Shox2*-mediated genetic modules in SAN development dictated by different Shox2 levels.

### Deletion of *Nkx2-5* in *Shox2* mutant background rescues the pacemaking cell fate and ameliorates the impaired physiological function of the SAN

**Fig. 2:**
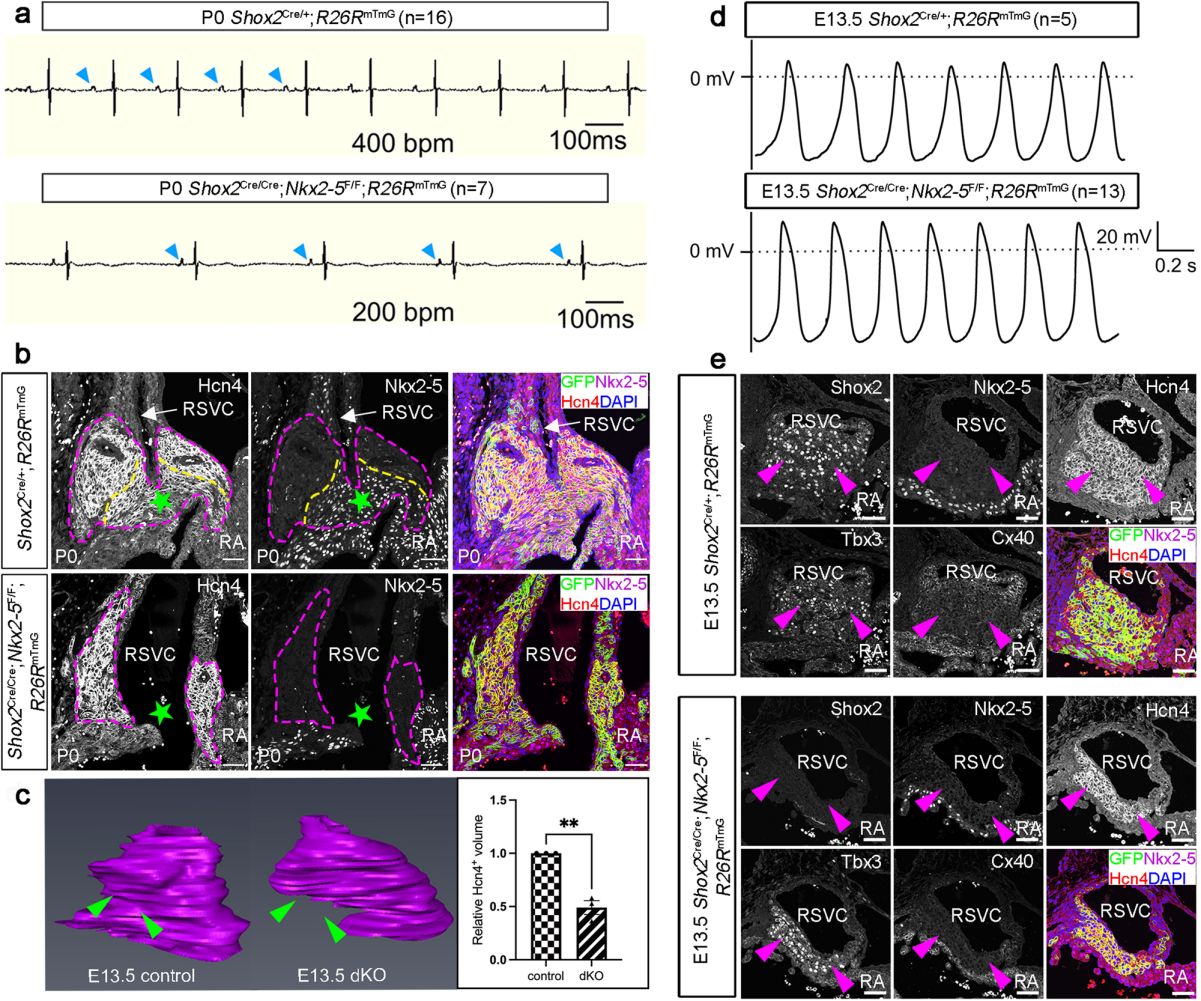
*Shox2*-*Nkx2-5* double mutant (dKO) mice display a hypoplastic SAN accompanied by bradycardia but retain the pacemaking cell fate. **a** Surface ECG recordings reveal normal P waves and regular rhythms but bradycardia in P0 *Shox2*^Cre/Cre^;*Nkx2-5*^F/F^;*R26R*^mTmG^ mice, compared with *Shox2*^Cre/+^;*R26R*^mTmG^ mice. Blue arrowheads point to P waves. **b** Immunostaining shows the absence of the Nkx2-5^+^ SAN junction structure and reduced size of the SAN head in P0 *Shox2*^Cre/Cre^;*Nkx2-5*^F/F^;*R26R*^mTmG^ mice, compared with *Shox2*^Cre/+^;*R26R*^mTmG^ mice. The SAN region is marked by the magenta dash-lines, and the yellow dash-lines separate the SAN head from the SAN junction (green stars). **c** 3D reconstructions of the SAN (based on Hcn4 immunostaining) in E13.5 *Shox2*^Cre/+^;*R26R*^mTmG^ and *Shox2*^Cre/Cre^;*Nkx2-5*^F/F^;*R26R*^mTmG^ mice (*n* = 3 for each genotype), respectively, reveal the absence of the SAN junction (green arrowheads) and dramatically reduced size of the entire SAN in the mutants, as confirmed by statistical analysis. **d** Typical SAN AP configurations were recorded in GFP^+^ cells from the SAN in E13.5 *Shox2*^Cre/Cre^;*Nkx2-5*^F/F^;*R26R*^mTmG^ mice (*n* = 13), which were comparable to that seen in the SAN cells from E13.5 *Shox2*^Cre/+^;*R26R*^mTmG^ mice (*n* = 5). **e** Immunostaining shows colocalization of Shox2, Nkx2-5, Hcn4, Tbx3, and Cx40 in the SAN head (magenta arrowheads) of E13.5 *Shox2*^Cre/+^;*R26R*^mTmG^ and *Shox2*^Cre/Cre^;*Nkx2-5*^F/F^;*R26R*^mTmG^ mice. Similar to *Shox2*^Cre/+^;*R26R*^mTmG^ control mice, deletion of *Nkx2-5* in the SAN of *Shox2* mutants (*Shox2*^Cre/Cre^;*Nkx2-5*^F/F^;*R26R*^mTmG^) retains the expression of the SAN markers. The presented data are mean ± SEM. Statistical analysis was performed with Student’s *t*-test (two-tailed, **P < 0.01). RA right atrium, dKO *Shox2*^Cre/Cre^, *Nkx2-5*^F/F^; RSVC right superior vena cava. Bar: 50 μm.

We have previously reported that *Shox2* maintains the pacemaker program in the Nkx2-5^+^ junction domain of the SAN by antagonizing *Nkx2-5* transcriptional output^27^, we therefore asked whether rebalancing the Shox2-Nkx2-5 antagonism can reset the original pacemaking cell fate in the developing SAN. We took advantage of the compound *Shox2*^Cre/Cre^;*Nkx2-5*^F/F^ alleles to delete *Nkx2-5* in the *Shox2* null background^29,31^. In contrast to the embryonic lethality of *Shox2*^Cre/Cre^ mice at mid-gestation due to cardiac defects, *Shox2*^Cre/Cre^;*Nkx2-5*^F/F^ mice (dKO) survived to postnatal day 0 (P0), indicating amelioration of the impaired physiological function of the SAN. Surface electrocardiogram (ECG) measurements on P0 mice showed regular but slower cardiac rhythm and the normal P waves in the dKO mice as compared to controls (*n* = dKO: 7/7; WT: 16/16) (Fig. 2a). Additionally, whole-cell patch-clamp recording on cells derived from the *Shox2*^+^ population in the SAN of E13.5 dKO mice showed typical AP configurations comparable to controls (Fig. 2d; Supplementary Fig. 2; Supplementary Data 1). Immunohistochemistry and in situ hybridization assays showed that, unlike the lack of Tbx3 and Hcn4 expression in *Shox2* null mice^22^, dKO mice retained the expression of SAN markers, including Tbx3, Hcn4 (Fig. 2e). This observation strongly implies the preservation of the pacemaking cell fate in dKO mice. Morphometric analyses on the SAN of dKO and *Shox2* null mice (Supplementary Fig. 1b–d) revealed that the SAN morphology in dKO mice appeared comparable to that in *Shox2* null mice^22,23^. However, upon closer inspection, we discovered that the Hcn4 expression in the SAN head domain (as opposed to the junction domain demarcated by *Nkx2-5* expression) of dKO mice was notably restored (Fig. 2b; Supplementary Fig. 1b–d), following the deletion of *Nkx2-5* in the *Shox2*-null background. This result suggests that the dKO mice retain the pacemaking cell fate by developing a hypoplastic SAN lacking the junction domain (Fig. 2c; Supplementary Data 1). Given previous reports indicating that the structural characteristics of the microenvironment surrounding pacemaking cells in the SAN can impact their function through local mechanics^32^, the sinus bradycardia ECG defects observed in dKO mice (Fig. 2a) may be attributed to the altered SAN morphology (hypoplasia). Above all, these observations demonstrate that rebalancing Shox2-Nkx2-5 antagonism by deletion of *Nkx2-5* in *Shox2*-null background rescues the pacemaking cell fate and ameliorates the impaired SAN physiological function but has no impact on SAN morphogenesis observed in *Shox2*^-/-^ mice. The exclusive function of Shox2-Nkx2-5 antagonistic mechanism in the regulation of SAN cell fate, but not in SAN morphogenesis, suggests that there are alternative genetic programs utilized by *Shox2* to control SAN morphogenesis.

### Single-cell transcriptomic analysis confirms the rescued pacemaking cell fate of the dKO SAN

**Fig. 3:**
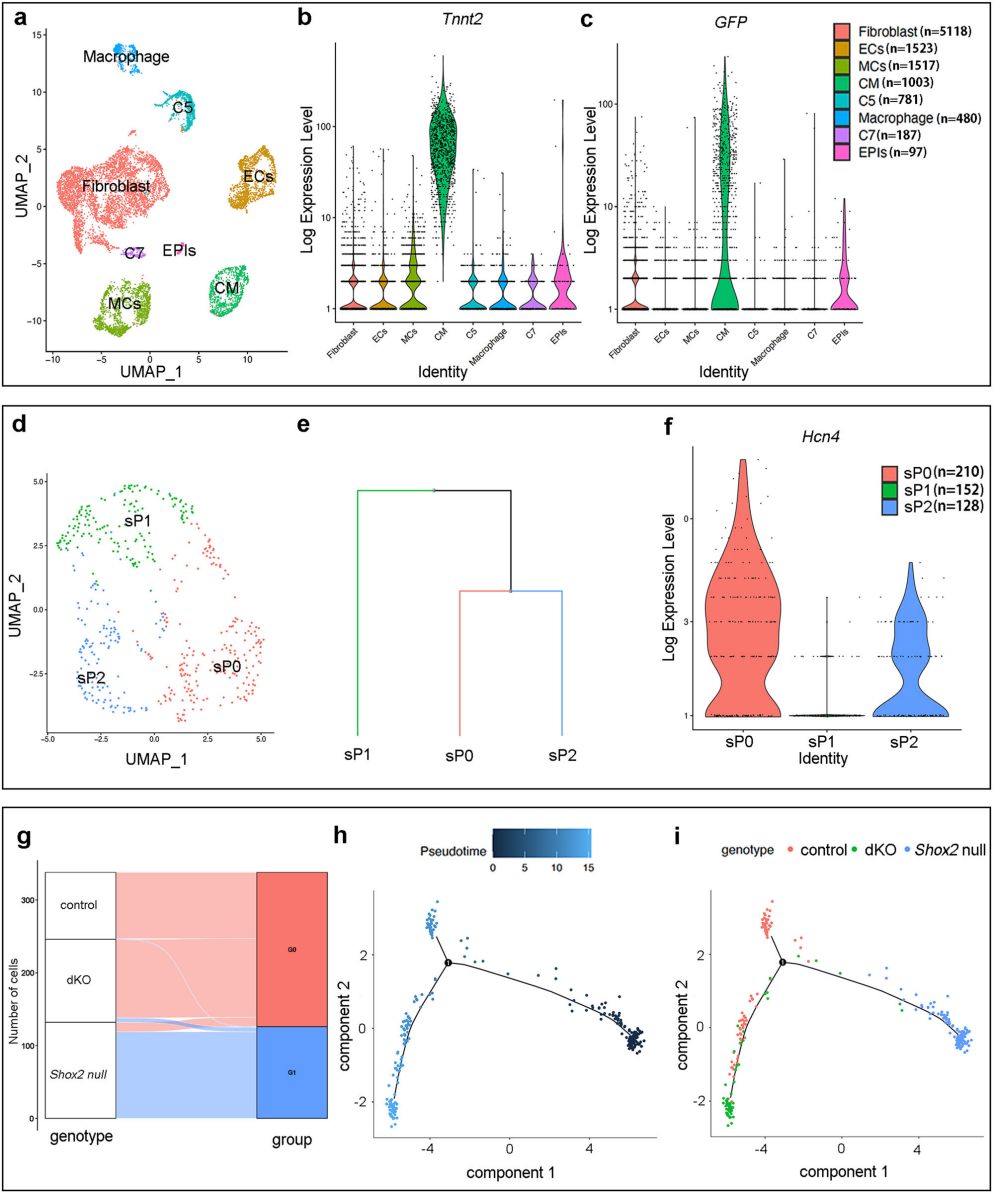
ScRNA-seq identifies transcriptomic profile of dKO SAN cells comparable to controls. **a** UMAP visualization of the eight clusters from the SAN and its adjacent atrial cells of E13.5 *Shox2*^Cre/+^;*R26R*^mTmG^ (control), *Shox2*^Cre/Cre^;*R26R*^mTmG^ (*Shox2* null), and *Shox2*^Cre/Cre^;*Nkx2-5*^F/F^;*R26R*^mTmG^ (dKO) mice. **b**, **c** VlnPlot shows the expression of *Tnnt2* and *GFP* probability distributions across eight clusters. **d**–**f** Analysis of three sub-populations from *Shox2*^+^ (represented by GFP^+^) cardiomyocytes defines sP0 and sP2 as the SAN cells. **g** Sankey Plot showing the SAN cells of control, *Shox2* null, and dKO reclassified into two groups. **h**, **i** Pseudotemporal ordering of SAN cells reveals that *Shox2* null cells exhibited the pseudo-start state, while the control and dKO cells displayed the pseudo-end state. Cells are colored by the genotype of samples (**i**). Cells with dark color represent the pseudo-start, and the bright color represent the pseudo-end (h). CM cardiomyocyte, EC endothelial cells, MC mesothelial cells, EPI epithelial cells, C5, C7 cluster number 5, 7.

The rescued pacemaking cell fate but not morphology of the SAN in dKO mice as well as the failure of SAN formation in the *Shox2*-null mice provided a unique model (Supplementary Table 2) for us to investigate how *Shox2* interacts with *Nkx2-5* to separately safeguard the pacemaker fate and regulate the SAN morphogenesis. To do so, we compounded three genetically modified alleles to generate *Shox2*^Cre/Cre^;*Nkx2-5*^F/F^;*R26R*^mTmG^ (dKO), *Shox2*^Cre/Cre^;*R26R*^mTmG^ (*Shox2* null), and *Shox2*^Cre/+^;*R26R*^mTmG^ (control) mice, and performed single-cell RNA-seq (scRNA-seq) on *Shox2*^+^ cells (labeled by GFP) from the SAN of E13.5 mice with these three different genotypes (control, *Shox2*-null, and dKO). The datasets obtained were subjected together to unsupervised clustering, resulting in 8 distinct clusters, including Fibroblast, endothelial cells, mesothelial cells (MCs), cardiomyocytes (CM), Macrophages, and epithelial cells (EPIs)^28,33–39^ (Fig. 3a). Based on the expression of *Tnnt2* and *GFP*, we were able to identify a *Shox2*^+^ cardiomyocyte population (Fig. 3b, c). The *Shox2*^+^ cardiomyocytes were then further distinguished into three sub-populations (sP0, sP1, and sP2) (Fig. 3d; Supplementary Fig. 3a). While sP0 and sP2 showed the closest phylogenic distance and expressed pan-SAN marker *Hcn4*^40^ (Fig. 3e, f), these two sub-populations were defined as pacemaker cells, the sP1 sub-population seemed likely the residual venous valve cells, as they have similar transcriptome with the venous valve we reported previously^28^. Further unsupervised sub-clustering reclassified the pacemaker cells into two groups, with the control and dKO pacemaker cells falling in group 0 (G0) and *Shox2*-null pacemaker cells in group 1 (G1) (Fig. 3g; Supplementary Fig. 3b, b’). These results indicate that the control and dKO cells share a high transcriptomic similarity that deviates from that of *Shox2-*null cells. To infer the cellular states of the pacemaker cells, we used Monocle2^41^ to pseudo-temporally order the pacemaker cells along a developmental trajectory to the differentiated pacemaking cells. Pseudotime trajectory analysis showed that while the control and dKO samples exhibit the most differentiated cellular state, *Shox2-*null sample displays the most primitive and under-differentiated cellular state (Fig. 3h, i; Supplementary Fig. 3c). This is evidenced by the increased expression of several widely recognized cardiac progenitor cell markers^42^, including *Abcg2*. *Abcg2*, which serves as the molecular marker for identifying side population progenitor cells in multiple adult tissues^43^, including the adult heart^44^, is also documented to be transiently expressed during the early stages of heart development^45^. Thus, the pacemaker cell fate-oriented differentiation process appears unaffected in the dKO cells but is blocked at a progenitor state in the *Shox2-*null cells, as compared to the control group. These results are in line with the rescued pacemaking cell fate in the dKO mice as shown above, providing a solid foundation for further analyses.

**Fig. 4:**
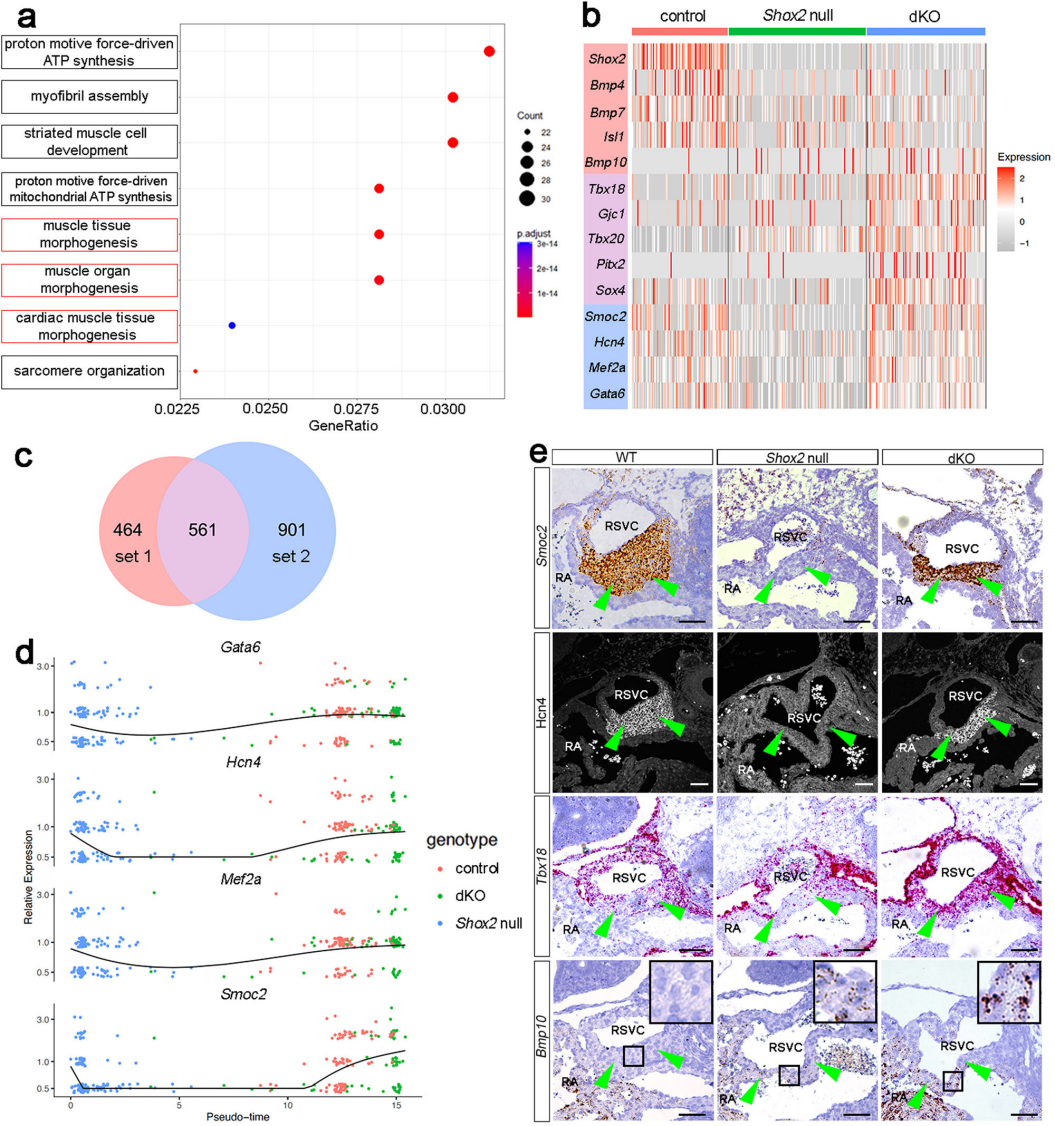
Validation of selected transcription targets of Shox2 involved in SAN morphogenesis v.s. pacemaking cell fate determination. **a** Go analysis on the DEGs between control and dKO reveals GO terms associated with heart development. **b** Heatmap shows the genes involved in the determination of the pacemaking cell fate and SAN morphogenetic regulation across the three samples. **c** Venn diagram depicts the number of overlap genes between pacemaking cell fate determination and SAN morphogenetic regulation. **d** Pseudotime analysis unravels elevated expression of *Gata6*, *Hcn4*, *Mef2a*, and *Smoc2* along pseudotime trajectory. **e** In situ hybridization and immunostaining verified the expression of *Smoc2*, *Tbx18*, *Bmp10*, and Hcn4 in the SAN of E13.5 control, *Shox2* null, and dKO mice. RA right atrium, RSVC right superior vena cava. Bar: 50 μm.

With the distinct physiological and morphological traits of the SAN in the three types of mice with distinct genetic background mentioned above, we reasoned that comparative analyses of the transcriptomic profiles between different sample would allow us to segregate the morphogenetic regulatory factors that are downstream of *Shox2* from the pacemaking cell fate determination factors (Supplementary Table 2). To identify genes that are potentially involved in SAN morphogenesis, we compared the transcriptomic profiles between the dKO and control mice using Seurat^46,47^. We identified 1025 genes that are differentially expressed between the dKO and control mice (set 1). Gene ontology (GO) analysis on the set 1 differentially expressed genes (DEGs) revealed the enrichment of morphogenesis-associated GO terms, such as muscle tissue morphogenesis, muscle organ morphogenesis, and cardiac muscle tissue morphogenesis (Fig. 4a). In an attempt to identify genes that are involved in SAN cell fate determination, we then compared the transcriptomes between the dKO and *Shox2* null samples and identified 1462 DEGs (set 2). Conversely, the identified set 2 DEGs included genes that are known to participate in pacemaking cell fate regulation, such as *Hcn4*, *Smoc2*, and *Gata6*^48^. These results suggest the feasibility of segregating the SAN morphogenetic regulatory factors from the pacemaking cell fate determination factors by comparative analyses on the transcriptomic profiles of the three samples. To segregate them, we then intersected the DEGs in set 1 and set 2 and found out that except 561 genes that were shared by these two sets, there are 464 genes that are exclusively presented in set 1, and 901 genes that were found exclusively in set 2 (Fig. 4c). These two gene sets are thus potentially involved in the segregation of SAN morphogenesis from cell fate determination. Heatmap analysis confirmed the differential expression of these genes (Fig. 4b). Furthermore, the differential expression of several selected genes including *Hcn4*, *Smoc2*, *Bmp10*, *Gata6*, *Mef2a*, and *Tbx18* was further confirmed by pseudotime trajectory analysis and by in situ hybridization and immunostaining assays (Fig. 4d, e). Interestingly, although it was shown previously that *Pitx2* and *Tbx20* act upstream of *Nkx2-5*^22,30,49,50^, we observed ectopic expression of *Pitx2* and *Tbx20* in the scRNA-seq profile of the dKO SAN (Fig. 4b), suggesting that the Shox2-Nkx2-5 antagonistic mechanism has a negative feedback effect on the upstream regulator genes, which warrants future investigation. Above all, our unique genetically modified mouse lines allowed us to segregate genes involved in the regulation of morphogenesis from those involved in cell fate determination downstream from Shox2 and Nkx2-5 in SAN development.

### ChIP-seq analysis reveals that the segregation of SAN morphogenesis from cell fate determination attributes to a functional interplay between Shox2 and Nkx2-5

**Fig. 5:**
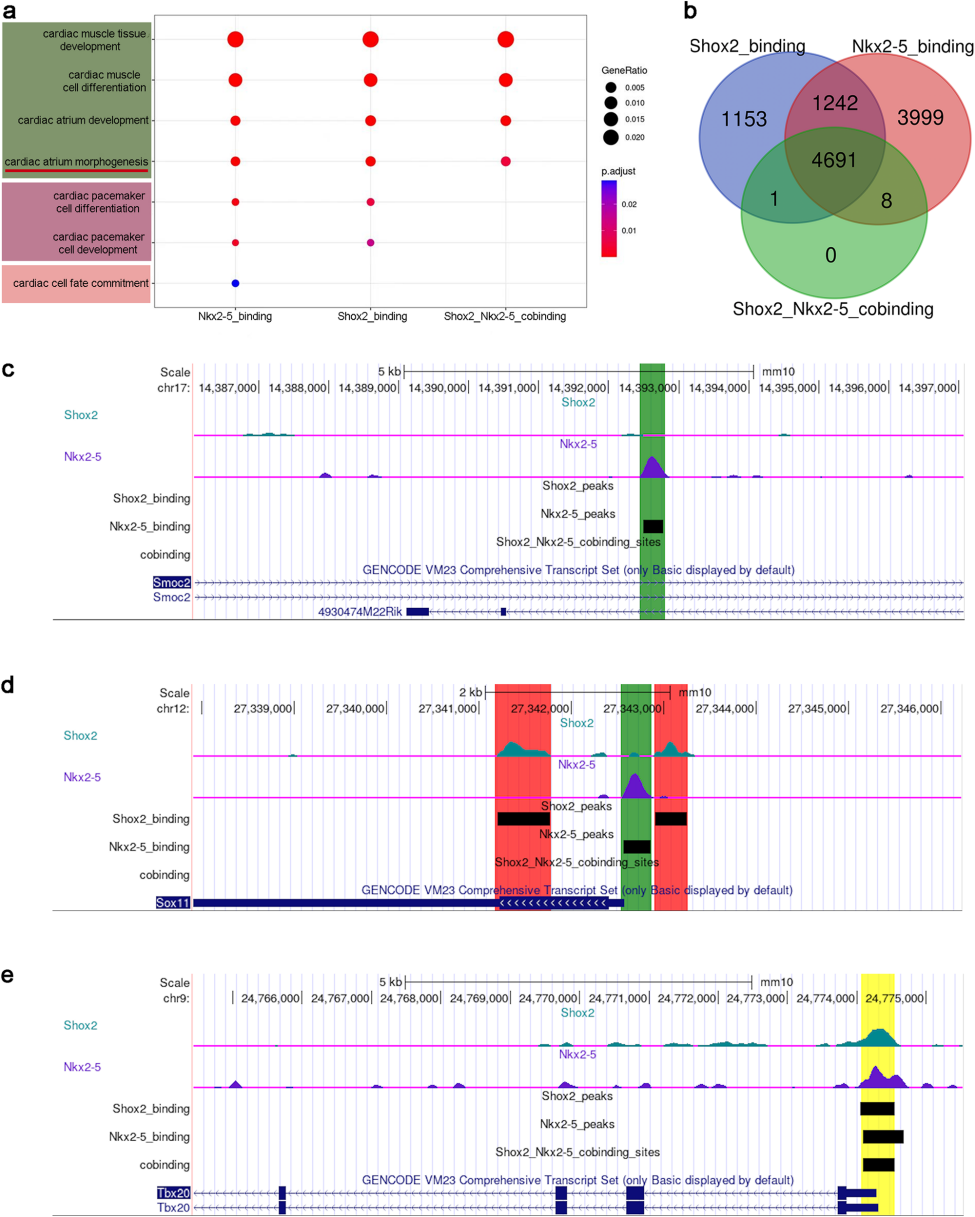
Integrative analyses identify direct targets of Shox2 and Nkx2-5 involved in the segregation of pacemaking cell fate commitment, safeguarding and SAN morphogenesis. **a** Functional categorization of Shox2 and Nkx2-5 binding genes. **b** Venn diagram depicts the number of overlapped genes between Shox2 binding, Nkx2-5 binding and Shox2-Nkx2-5 co-binding genes. **c**–**e** UCSC genome browser track displaying the binding patterns on the representative genes that are potentially involved in the segregation of pacemaking cell fate commitment, safeguarding, and SAN morphogenesis.

Since the two gene sets are identified from *Shox2* and *Nkx2-5* mutant mice, to verify the identified genes and to further understand how Shox2 interacts with Nkx2-5 to regulate SAN morphogenesis and cell fate determination separately, we re-analyzed our published Shox2 and Nkx2-5 ChIP-seq datasets on the developing heart^27^. As outlined in our previous report^27^, Shox2 and Nkx2-5 exhibit genome-wide co-occupancy (Supplementary Fig. 4a). However, characterization of binding peak distribution patterns for Shox2 and Nkx2-5 unveils that Shox2 exhibits a higher affinity for binding to the promoter regions (82.65%) compared to Nkx2-5 (64.81%). Conversely, Nkx2-5 displays a stronger preference for binding to the distal cis-regulatory elements, such as distal intergenic regions (Supplementary Fig. 4b). Intersection of Shox2 and Nkx2-5 binding peaks reveals that the co-binding regions of Shox2-Nkx2-5 are predominantly located within the promoter regions, similar to the distribution of Shox2 peaks (Supplementary Fig. 4c). Interestingly, when functionally categorizing the target genes associated with Shox2 binding, Shox2-Nkx2-5 co-binding, and Nkx2-5 binding peaks, it becomes evident that these genes play distinct roles in cardiac-related GO terms. Specifically, genes bound by Shox2, including those that are co-bound by Shox2-Nkx2-5, were observed to contribute to pacemaker cell differentiation and SAN morphogenesis (Fig. 5a). On the other hand, the genes exclusively bound by Nkx2-5 were linked to the cardiac cell fate commitment (Fig. 5a). This result aligns with Nkx2-5’s binding preference and is consistent with our previous study, where we highlighted its role in defining a subpopulation of pacemaker cells^27^. More interestingly, only the genes with individual binding of Shox2 and Nkx2-5, rather than co-binding of Shox2-Nkx2-5, are classified within the context of cardiac pacemaker cell development and differentiation (Fig. 5a), implicating that the segregation of Shox2’s morphogenetic regulatory function from its cell fate safeguarding role arises due to combinational interactions between Shox2 and Nkx2-5. These results not only reinforce our segregated gene outcomes, but also underscore the significance of delving deeper into the intricate mechanisms of Shox2-Nkx2-5 antagonism. Therefore, we conducted an intersection between the list of 3999 genes displaying exclusive Nkx2-5 binding (Fig. 5b) and the gene set 2 (Fig. 4c), which is potentially involved in cell fate determination. This analysis aimed to pinpoint genes associated with SAN cell fate commitment, yielding a list of 333 genes directly targeted by Nkx2-5. Notably, this list of genes encompasses those that have been previously implicated in cardiac cell fate determination, such as *Smoc2*^48^ (Fig. 5c) and *Taz*^51^ (Supplementary Data 2). To distinguish genes involved in safeguarding cell fate from those associated with morphogenesis, we performed further intersections on the following gene groups: firstly, the group of 1242 genes with both Shox2 and Nkx2-5 binding, but no Shox2-Nkx2-5 co-binding; and the group of 4691 genes with all three binding patterns of Shox2 and Nkx2-5 (Fig. 5b). These intersections were performed with gene set 1, yielding a list of 105 genes possessing both Shox2 and Nkx2-5 binding sites but lacking co-binding sites (Fig. 5d, Supplementary Data 3), and a separate list of 429 genes with all three binding patterns (Fig. 5e, Supplementary Data 4). While the 429 genes with all three binding patterns are potentially involved in SAN morphogenesis, we assumed that these 105 genes with both Shox2 and Nkx2-5 binding but no co-binding sites act as safeguarding factors. Interestingly, among these 105 genes, we found the DEGs that were shown in the regulation of both cell fate determination and morphogenesis through scRNA-seq analysis, including *Tbx18*, *Pitx2* (Supplementary Fig. 4d, e), and *Sox11*(Fig. 5d). This further substantiates the notion that the segregation during SAN development results from the functional interplay between Shox2 and Nkx2-5. Taken together, our integrative analyses of ChIP-seq and scRNA-seq datasets verified and identified genes that are distinctly involved in the regulation of pacemaking cell fate commitment, safeguarding and SAN morphogenesis, respectively. However, since the ChIP-seq assays were performed on the whole atrium of the heart that contains the SAN, atrial myocardial cells, and non-myocardial cells, while the scRNA-seq was done on the SAN cells, further studies are needed to verify our identified genes.

In this study, we have uncovered that the essential role of *Shox2* in SAN morphogenesis is uncoupled from its function in promoting pacemaking cell fate. We have unraveled that the altered pacemaking cell fate in the SAN of *Shox2* mutant mice can be reset by rebalancing Shox2-Nkx2-5 antagonism in *Shox2* and *Nkx2-5* dKO mice, despite the presence of a hypoplastic SAN. A morphologically integrated SAN is essential for mammals to retain the dominant pacemaker site in a proper position^52^. Theoretical studies and empirical evidence point toward a morphogenetic program for the SAN that is evolved independently from the pacemaker program^12,53,54^. In this study, we showed that despite the presence of well-differentiated pacemaking cells in the SAN, the dKO mice nevertheless displayed bradycardia symptom, which was also seen in *Shox2* null mutants, indicating the impact of the altered morphology on the physiological function of the SAN. Our scRNA-seq on the SAN cells from those unique mouse lines allow us to establish a *Shox2* and *Nkx2-5* regulated gene expression profile exclusively implicated in the regulation of SAN morphogenesis along with a gene expression profile specifically involved in pacemaking cell determination and differentiation, which warrants future investigation and verification. These gene expression profiles would also provide candidate genes for the etiology of congenital and acquired abnormalities of the SAN and potential targets for therapeutic intervention of sinus node dysfunction.

## Methods

### Animal models, embryos, histology, immunofluorescence, and in situ hybridization

The generation and genotyping protocols of *Shox2*^Cre^, *Shox2*^HA-Neo^*, Nkx2.5*^F/F^, and *R26R*^mTmG^ mice have been described previously^29–31,55^. The animal experiments in this study were approved by the Institutional Animal Care and Use Committee of Tulane University and by the Animal Ethic Committee of Fujian Normal University. We have complied with all relevant ethical regulations for animal use.

Timed pregnant mice were euthanized to collect embryos and embryonic hearts, which were fixed in 4% paraformaldehyde (PFA) at 4 °C overnight, paraffin-embedded, and sectioned at 8 μm for histology and immunofluorescent staining as described previously^28^. The primary antibodies used in this study were: anti-Shox2 (homemade; 1:500), anti-Nkx2-5 (sc-8697, Santa Cruz; 1:500), anti-Tbx3 (sc-17871, Santa Cruz; 1:500), anti-Hcn4 (ab32675, Abcam; 1:500), anti-Cx40 (sc-20466, Santa Cruz; 1:250), anti-GFP (sc-9996, Santa Cruz; 1:500). The secondary antibodies were all purchased from Life Technologies and used at 1:1000 dilution: donkey-anti rat (A21208, A21209), donkey-anti rabbit (A31573), donkey-anti mouse (A21202, A31571), donkey-anti goat (A32849, A11058, A11055).

In situ hybridization was performed with the RNAscope 2.5 HD Reagent Kit Brown (Advanced Cell Diagnostics, 322300) according to the manufacturer’s instructions. Probe informations are as below: *Bmp10* probe (RNAscope® Probe - Mm-Bmp10, 415921), *Smoc2* probe (RNAscope® Probe - Mm-Smoc2, 318541), *Tbx18* probe (RNAscope® Probe- Mm-Tbx18-C2, 515221-C2). All experiments were repeated at least three times with consistent results.

### Surface ECG, whole-cell patch-clamp recording, and scRNA-seq

Surface ECG recording of P0 mice were conducted as reported previously^28^. On the morning of the birthday, entire litter from *Shox2*^Cre/+^;*Nkx2-5*^F/F^ female crossed to *Shox2*^Cre/+^;*Nkx2-5*^F/F^ male was collected and each pup was subjected to ECG measurement.

For whole-cell patch-clamp recording, embryonic hearts were collected from E13.5 *Shox2*^Cre/+^;*R26R*^mTmG^ and *Shox2*^Cre/Cre^;*Nkx2.5*^F/F^;*R26R*^mTmG^ mice, respectively, and the GFP^+^ SAN was dissected out under a fluorescent dissecting microscope. Tissues were dissociated into single cells that were plated in culture and subjected to whole-cell patch-clamp recording as previous study^28^.

For single-cell RNA-seq (scRNA-seq), the GFP^+^ SAN and residual adjacent atrial tissues from E13.5 *Shox2*^Cre/+^;*R26R*^mTmG^, *Shox2*^Cre/Cre^;*R26R*^mTmG^, and *Shox2*^Cre/Cre^;*Nkx2.5*^F/F^;*R26R*^mTmG^ mice were isolated under a stereo fluorescent microscope. Tissues were subjected to single-cell dissociation, library preparation, sequencing, and bioinformatic analyses, as described in detail previously^28^.

### RT-qPCR analyses

For quantitative reverse transcription polymerase chain reaction (RT-qPCR), the atrium was isolated from E12.5 WT, *Shox2*^HA-Neo/+^, and *Shox2*^HA-Neo/Cre^ mice, and subjected to RNA extraction (E.Z.N.A.® Total RNA Kit I, R6834-02). The RNAs were subsequently reversely transcribed into complementary DNAs (cDNAs) (PrimeScript™ RT reagent Kit, RR037A). SYBR green and gene-specific primers were used and transcript levels were examined using a Thermo Scientific PikoReal Real-Time PCR System. Primer details are as follows: *Shox2* (F, 5′-ACCAATTTTACCCTGGAACAAC-3′; R, 5′-TCGATTTTGAAACCAAACCTG-3′), *GAPDH* (F, 5′-ATCAAGAAGGTGGTGAAGCAG-3′; R, 5′-GAGTGGGAGTTGCTGTTGAAGT-3′). Differences in the RT-qPCR were analyzed using Student’s *t*-test in GraphPad Prism7; results are presented as mean ± SEM. *P* < 0.05 was considered significant.

### 3D reconstruction

For 3D reconstruction, tissues were processed into consecutive sections at 10 µm, stained by anti-Hcn4 antibodies, imaged, and loaded into Amira 6.0.1, in which subsequent alignment, segmentation, and 3D model generation were performed, as reported previously^16,28^.

### Statistics and Reproducibility

All experiments were repeated at least three times unless specifically indicated. All data were statistically analyzed by GraphPad Prism 7. The data were presented as mean ± standard error of the mean (SEM). Statistical analysis was performed using Student’s *t*-test. Results were considered statistically significant at *P* < 0.05.

### Reporting summary

Further information on research design is available in the Nature Portfolio Reporting Summary linked to this article.

### Supplementary information

Supplementary Information

Description of Additional Supplementary Files

Supplementary Data 1

Supplementary Data 2

Supplementary Data 3

Supplementary Data 4

Reporting Summary

## Data Availability

ScRNA-Seq data have been deposited to Gene Expression Omnibus (GEO) with access number GSE143997.

## References

[1] Boyett MR, Honjo H, Kodama I (2000). The sinoatrial node, a heterogeneous pacemaker structure. Cardiovasc. Res..

[2] Liu J, Dobrzynski H, Yanni J, Boyett MR, Lei M (2007). Organisation of the mouse sinoatrial node: structure and expression of HCN channels. Cardiovasc. Res..

[3] Opthof T (1988). The mammalian sinoatrial node. Cardiovasc Drugs Ther..

[4] Christoffels VM, Smits GJ, Kispert A, Moorman AF (2010). Development of the pacemaker tissues of the heart. Circ. Res..

[5] Moorman AF, Christoffels VM (2003). Cardiac chamber formation: development, genes, and evolution. Physiol. Rev..

[6] Liu H (2011). Functional redundancy between human SHOX and mouse Shox2 genes in the regulation of sinoatrial node formation and pacemaking function. J. Biol. Chem..

[7] Hu W, Xin Y, Zhao Y, Hu J (2018). Shox2: the role in differentiation and development of cardiac conduction system. Tohoku J. Exp. Med..

[8] Van Mierop L, Gessner IH (1970). The morphologic development of the sinoatrial node in the mouse. Am. J. Cardiol..

[9] Virágh S, Challice C (1977). The development of the conduction system in the mouse embryo heart: II. Histogenesis of the atrioventricular node and bundle. Dev. Biol..

[10] Christoffels VM (2006). Formation of the venous pole of the heart from an Nkx2–5–negative precursor population requires Tbx18. Circ. Res..

[11] Mommersteeg MT (2007). Molecular pathway for the localized formation of the sinoatrial node. Circ. Res..

[12] Wiese C (2009). Formation of the sinus node head and differentiation of sinus node myocardium are independently regulated by Tbx18 and Tbx3. Circ. Res..

[13] Van Mierop L (1967). Location of pacemaker in chick embryo heart at the time of initiation of heartbeat. Am. J. Physiol. Leg. Content.

[14] Munshi NV (2012). Gene regulatory networks in cardiac conduction system development. Circ. Res..

[15] van Eif VW (2019). Transcriptome analysis of mouse and human sinoatrial node cells reveals a conserved genetic program. Development.

[16] Sun C (2015). The short stature homeobox 2 (Shox2)-bone morphogenetic protein (BMP) pathway regulates dorsal mesenchymal protrusion development and its temporary function as a pacemaker during cardiogenesis. J. Biol. Chem..

[17] DiFrancesco D (2010). The role of the funny current in pacemaker activity. Circ. Res..

[18] Moosmang S (2001). Cellular expression and functional characterization of four hyperpolarization‐activated pacemaker channels in cardiac and neuronal tissues. Eur. J. Biochem..

[19] Santoro B, Tibbs GR (1999). The HCN gene family: molecular basis of the hyperpolarization‐activated pacemaker channels. Ann. N. Y. Acad. Sci..

[20] Frank DU (2012). Lethal arrhythmias in Tbx3-deficient mice reveal extreme dosage sensitivity of cardiac conduction system function and homeostasis. Proc. Natl Acad. Sci..

[21] Tang, W. C. *Cell Movement in Health and Disease* (eds Michael Schnoor, Lei-Miao Yin, Sean X. Sun) 151–157 (Academic Press, 2022).

[22] Espinoza-Lewis RA (2009). Shox2 is essential for the differentiation of cardiac pacemaker cells by repressing Nkx2-5. Dev. Biol..

[23] Blaschke RJ (2007). Targeted mutation reveals essential functions of the homeodomain transcription factor Shox2 in sinoatrial and pacemaking development. Circulation.

[24] Vedantham V (2015). New approaches to biological pacemakers: links to sinoatrial node development. Trends Mol. Med..

[25] Espinoza-Lewis RA (2011). Ectopic expression of Nkx2. 5 suppresses the formation of the sinoatrial node in mice. Dev. Biol..

[26] Kasahara H, Bartunkova S, Schinke M, Tanaka M, Izumo S (1998). Cardiac and extracardiac expression of Csx/Nkx2. 5 homeodomain protein. Circ. Res..

[27] Ye W (2015). A common Shox2–Nkx2-5 antagonistic mechanism primes the pacemaker cell fate in the pulmonary vein myocardium and sinoatrial node. Development.

[28] Li H (2019). Nkx2-5 defines a subpopulation of pacemaker cells and is essential for the physiological function of the sinoatrial node in mice. Development.

[29] Sun C, Zhang T, Liu C, Gu S, Chen Y (2013). Generation of Shox2‐Cre allele for tissue specific manipulation of genes in the developing heart, palate, and limb. Genesis.

[30] Wang J (2014). Pitx2-microRNA pathway that delimits sinoatrial node development and inhibits predisposition to atrial fibrillation. Proc. Natl Acad. Sci..

[31] Pashmforoush M (2004). Nkx2-5 pathways and congenital heart disease: loss of ventricular myocyte lineage specification leads to progressive cardiomyopathy and complete heart block. Cell.

[32] Henley T (2023). Local tissue mechanics control cardiac pacemaker cell embryonic patterning. Life Sci. Alliance.

[33] DeLaughter DM (2016). Single-cell resolution of temporal gene expression during heart development. Dev. cell.

[34] Khazen W (2005). Expression of macrophage‐selective markers in human and rodent adipocytes. FEBS Lett..

[35] Lee JH, Protze SI, Laksman Z, Backx PH, Keller GM (2017). Human pluripotent stem cell-derived atrial and ventricular cardiomyocytes develop from distinct mesoderm populations. Cell Stem Cell.

[36] Li G (2016). Transcriptomic profiling maps anatomically patterned subpopulations among single embryonic cardiac cells. Dev. cell.

[37] Souders CA, Bowers SL, Baudino TA (2009). Cardiac fibroblast: the renaissance cell. Circ. Res..

[38] Tarnawski L (2015). Integrin based isolation enables purification of murine lineage committed cardiomyocytes. PloS One.

[39] Vanlandewijck M (2018). A molecular atlas of cell types and zonation in the brain vasculature. Nature.

[40] Zappia L, Oshlack A (2018). Clustering trees: a visualization for evaluating clusterings at multiple resolutions. GigaScience.

[41] Trapnell C (2014). Pseudo-temporal ordering of individual cells reveals dynamics and regulators of cell fate decisions. Nat. Biotechnol..

[42] Iancu CB (2015). Molecular signatures of cardiac stem cells. Rom. J. Morphol. Embryol..

[43] Bunting KD (2002). ABC transporters as phenotypic markers and functional regulators of stem cells. Stem Cells.

[44] Pfister O (2008). Role of the ATP-binding cassette transporter Abcg2 in the phenotype and function of cardiac side population cells. Circ. Res.

[45] Martin CM (2004). Persistent expression of the ATP-binding cassette transporter, Abcg2, identifies cardiac SP cells in the developing and adult heart. Dev. Biol..

[46] Butler A, Hoffman P, Smibert P, Papalexi E, Satija R (2018). Integrating single-cell transcriptomic data across different conditions, technologies, and species. Nat. Biotechnol..

[47] Stuart T (2019). Comprehensive integration of single-cell data. Cell.

[48] van der Maarel, L. E., Postma, A. V. & Christoffels, V. M. Genetics of sinoatrial node function and heart rate disorders. *Dis. Model. Mech.* **16**, 10.1242/dmm.050101 (2023). 10.1242/dmm.050101 PMC10214849 37194974

[49] Stennard FA (2003). Cardiac T-box factor Tbx20 directly interacts with Nkx2-5, GATA4, and GATA5 in regulation of gene expression in the developing heart. Dev. Biol..

[50] Takeuchi JK (2005). Tbx20 dose-dependently regulates transcription factor networks required for mouse heart and motoneuron development. Development.

[51] Zheng, M. et al. Hippo-Yap Signaling Maintains Sinoatrial Node Homeostasis. *Circulation* **146**, 1694–1711 (2022) 10.1161/CIRCULATIONAHA.121.058777 PMC9897204 36317529

[52] Burkhard S, Van Eif V, Garric L, Christoffels VM, Bakkers J (2017). On the evolution of the cardiac pacemaker. J. Cardiovasc. Dev. Dis..

[53] Jensen B, Boukens BJ, Wang T, Moorman AF, Christoffels VM (2014). Evolution of the sinus venosus from fish to human. J. Cardiovasc. Dev. Dis..

[54] Ye W, Song Y, Huang Z, Zhang Y, Chen Y (2015). Genetic regulation of sinoatrial node development and pacemaker program in the venous pole. J. Cardiovasc. Dev. Dis..

[55] Muzumdar MD, Tasic B, Miyamichi K, Li L, Luo L (2007). A global double‐fluorescent Cre reporter mouse. Genesis.

