# Supplementary material for: Segregation of morphogenetic regulatory function of Shox2 from its cell fate guardian role in sinoatrial node development
Source: Commun Biol. 2024 Mar 29;7:385. doi: 10.1038/s42003-024-06039-2 (PMC10980793; doi:10.1038/s42003-024-06039-2)
Supplement: Supplementary file 1 — Supplementary Information [file 42003_2024_6039_MOESM1_ESM.pdf]

Supplementary Materials

Supplementary Tables

Supplementary Table 1. Summary of relative expression levels in compound *Shox2* alleles

Compound allele	*Shox2*$^{+/+}$	*Shox2*$^{HA\text{-}Neo/+}$	*Shox2*$^{Cre/+}$	*Shox2*$^{HA\text{-}Neo/HA\text{-}Neo}$	*Shox2*$^{HA\text{-}Neo/Cre}$	*Shox2*$^{Cre/Cre}$ (*Shox2* null)
Shox2 expression level	100%	65%	50%	30%	15%	0

Supplementary Table 2. Transcriptomic comparison analysis pipeline to segregate SAN cell fate determination and genes from morphogenesis genes

	SAN cell fate	SAN morphogenesis
Shox2$^{+/+}$(WT)	○	○
Shox2$^{Cre/Cre}$ (*Shox2* null)	✕	✕
Shox2$^{Cre/Cre}$;*Nkx2-5*$^{F/F}$(dKO)	○	✕

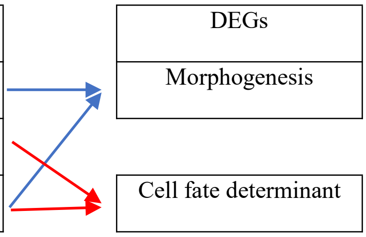

Supplementary Figures

Supplementary Figure 1. Coimmunostaining of Tnnt2, Hcn4, Nkx2-5, and Shox2 on the SAN of control, *Shox2* null, and dKO mice at E10.5 to E12.5.

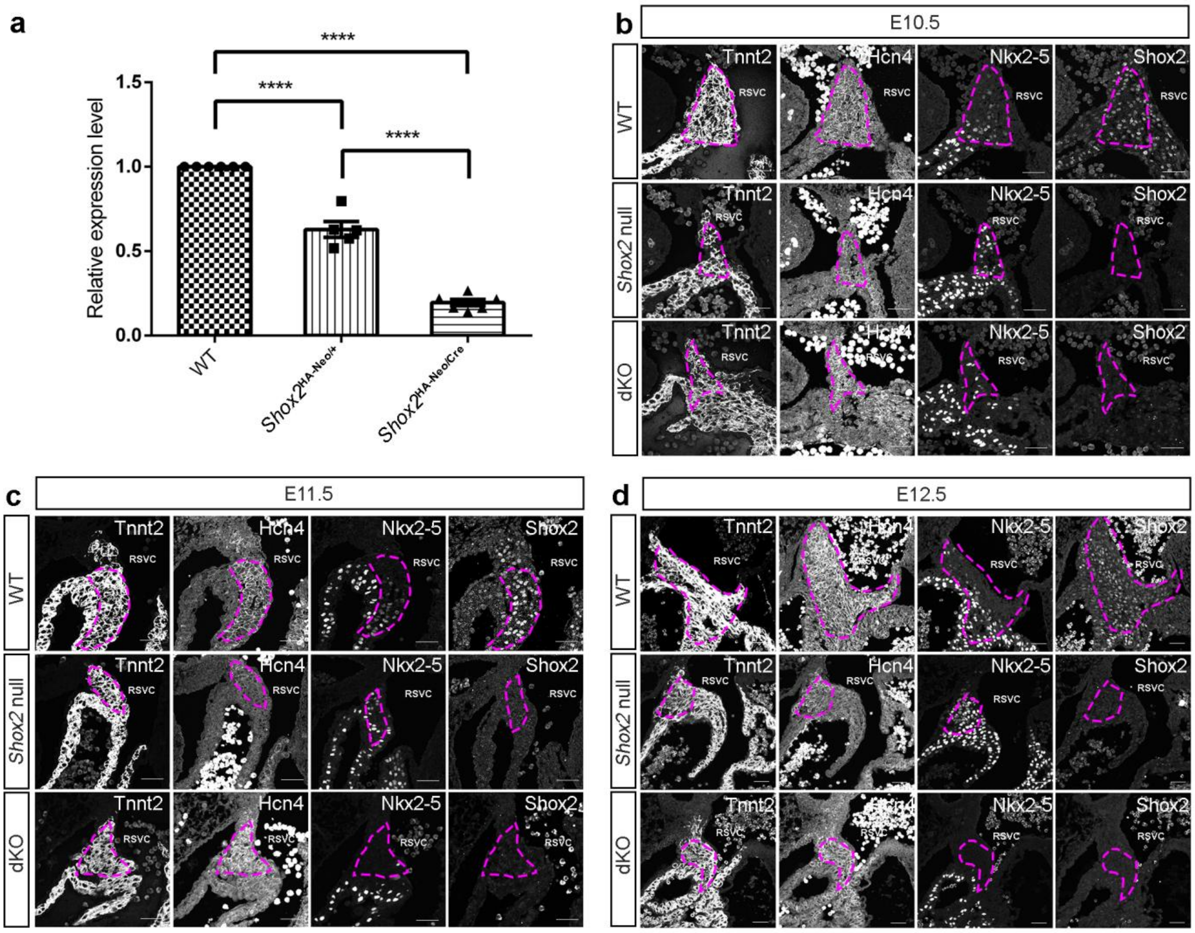

a RT-qPCR analyses shows reduction of *Shox2* expression in the atrium from E12.5 *Shox2*$^{\text{HA-Neo/+}}$ mice to around 65% and *Shox2*$^{\text{HA-Neo/Cre}}$ mice to 15% compared to wildtype controls. **b-d** Coimmunostaining of Tnnt2, Hcn4, Nkx2-5, and Shox2 on the SAN of control, *Shox2* null, and dKO mice at E10.5 to E12.5 reveals comparable SAN morphology between dKO and *Shox2* null mice. The SAN region is marked by the magenta dash-lines. Data are mean ± SEM. Statistical analysis was performed with Student's t-test (two-tailed, ****$P < 0.0001$). RSVC, right superior vena cava. Bar: 50 µm.

Supplementary Figure 2. Comparison of the AP parameters between the GFP$^+$ cells of the SAN from E13.5 control and dKO mice.

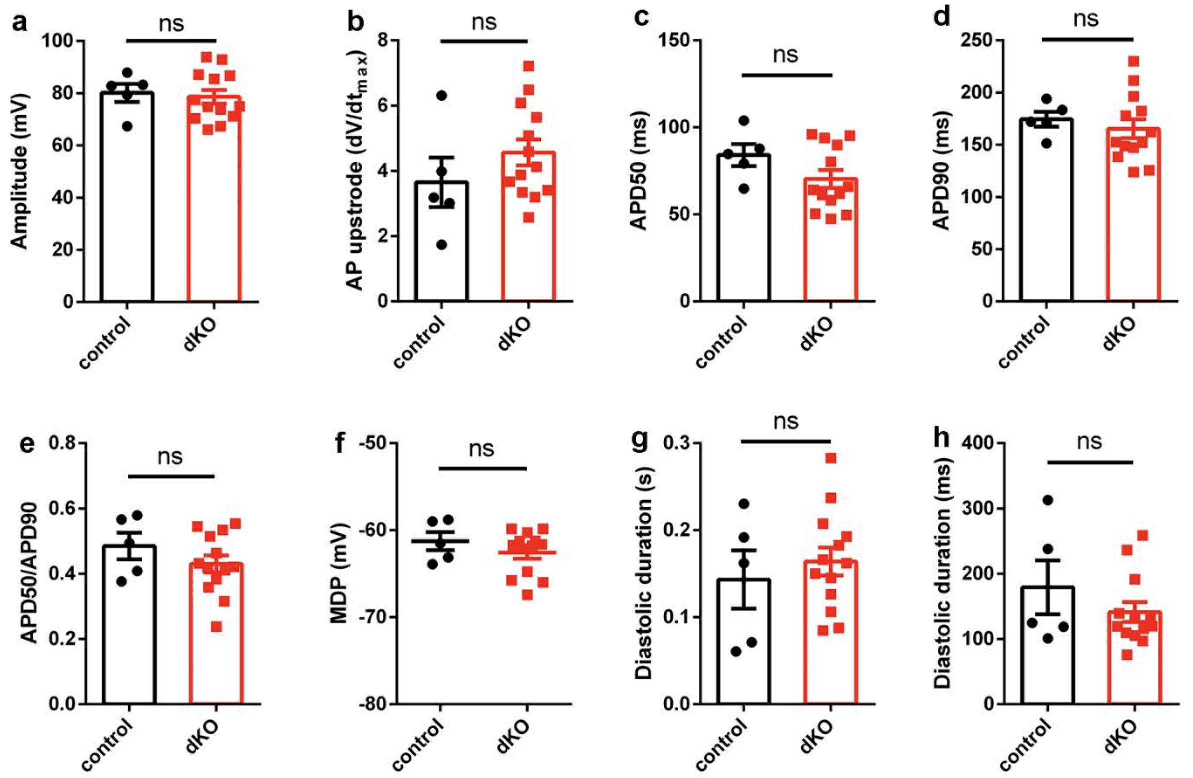

a-h Histograms show AP parameters between the GFP$^+$ cells of the SAN from control and dKO mice at E13.5, demonstrating comparable typical AP configurations between control and dKO mice. The presented data are mean ± SEM. Statistical analysis was performed with Student's *t*-test (two-tailed, ns: non-significant).

Supplementary Figure 3. Detailed analysis of sP0, sP1, and sP2.

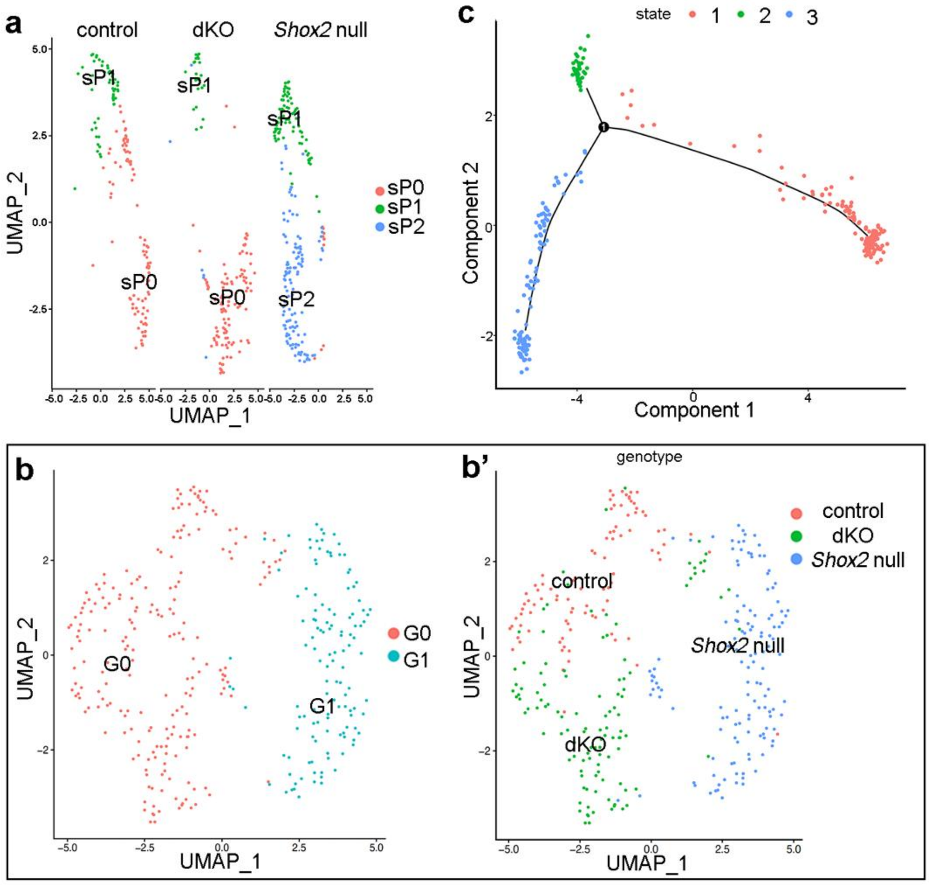

a Split view of the UMAP on the GFP^{+} CM cells of control, *Shox2* null, and dKO mice. **b, b'** Original dimplot images show the control and dKO pacemaker cells falling in group 0 (G0) and *Shox2*-null pacemaker cells in group 1 (G1). **c** Distinct states within the SAN cells from three samples along the pseudotime trajectory.

Supplementary Figure 4. Characterization of Shox2 and Nkx2-5 genome-wide binding sites in the heart.

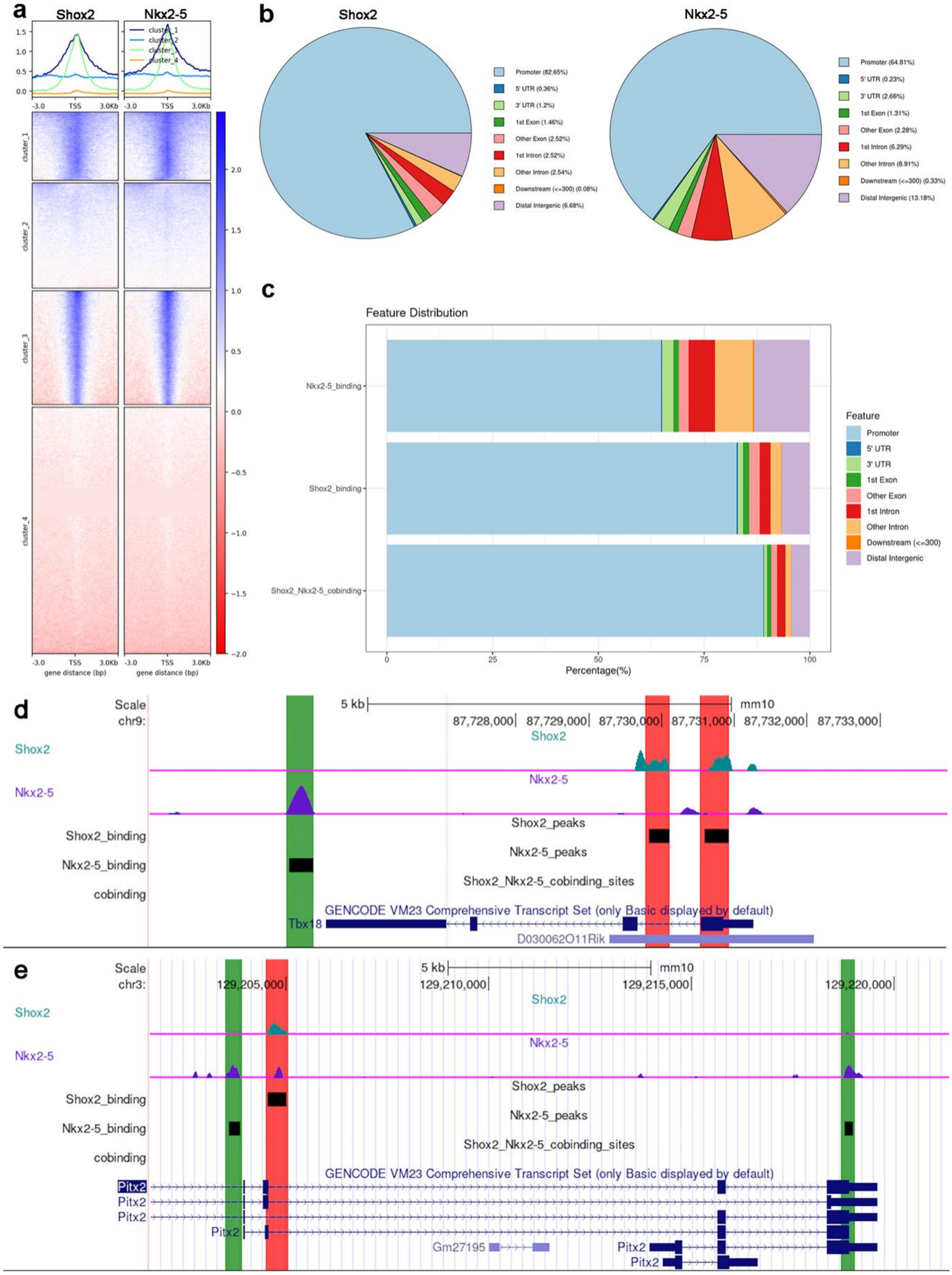

a Heatmaps show the genome-wide co-occupancy of Shox2 and Nkx2-5 in the heart. **b** Pie plots show the preferential binding of Shox2 in the promoter region than Nkx2-5. **c** Bar plots show the genomic

distributions of Shox2 specific, Nkx2-5 specific and Shox2-Nkx2-5 co-binding peaks. **d-e** UCSC genome browser track displaying the binding patterns of Shox2 and Nkx2-5 on *Tbx18* and *Pitx2*.
