# Supplementary material for: Segregation of morphogenetic regulatory function of Shox2 from its cell fate guardian role in sinoatrial node development
Source: Commun Biol. 2024 Mar 29;7:385. doi: 10.1038/s42003-024-06039-2 (PMC10980793; doi:10.1038/s42003-024-06039-2)
Supplement: Supplementary file 2 — Description of Additional Supplementary Files [file 42003_2024_6039_MOESM2_ESM.pdf]

Description of Additional Supplementary Files

File name: Supplementary Data 1

Description: The Source data for plots in the figures.

File name: Supplementary Data 2-4

Description: Genes that are respectively involved in SAN cell fate determination, cell fate safeguard and morphogenesis.
