# Supplementary material for: Segregation of morphogenetic regulatory function of Shox2 from its cell fate guardian role in sinoatrial node development
Source: Commun Biol. 2024 Mar 29;7:385. doi: 10.1038/s42003-024-06039-2 (PMC10980793; doi:10.1038/s42003-024-06039-2)
Supplement: Supplementary file 7 — Reporting Summary [file 42003_2024_6039_MOESM7_ESM.pdf]

nature portfolio

Corresponding author(s): Hua Li
COMMSBIO-23-1356D

Last updated by author(s): Feb 7, 2024

Reporting Summary

Nature Portfolio wishes to improve the reproducibility of the work that we publish. This form provides structure for consistency and transparency in reporting. For further information on Nature Portfolio policies, see our Editorial Policies and the Editorial Policy Checklist.

Statistics

For all statistical analyses, confirm that the following items are present in the figure legend, table legend, main text, or Methods section.

n/a	Confirmed	
☐	☒	The exact sample size (*n*) for each experimental group/condition, given as a discrete number and unit of measurement
☐	☒	A statement on whether measurements were taken from distinct samples or whether the same sample was measured repeatedly
☐	☒	The statistical test(s) used AND whether they are one- or two-sided<br>*Only common tests should be described solely by name; describe more complex techniques in the Methods section.*
☒	☐	A description of all covariates tested
☒	☐	A description of any assumptions or corrections, such as tests of normality and adjustment for multiple comparisons
☒	☐	A full description of the statistical parameters including central tendency (e.g. means) or other basic estimates (e.g. regression coefficient) AND variation (e.g. standard deviation) or associated estimates of uncertainty (e.g. confidence intervals)
☐	☒	For null hypothesis testing, the test statistic (e.g. *F*, *t*, *r*) with confidence intervals, effect sizes, degrees of freedom and *P* value noted<br>*Give P values as exact values whenever suitable.*
☒	☐	For Bayesian analysis, information on the choice of priors and Markov chain Monte Carlo settings
☒	☐	For hierarchical and complex designs, identification of the appropriate level for tests and full reporting of outcomes
☒	☐	Estimates of effect sizes (e.g. Cohen's *d*, Pearson's *r*), indicating how they were calculated

Our web collection on statistics for biologists contains articles on many of the points above.

Software and code

Policy information about availability of computer code

Data collection	No software was used.
Data analysis	Data were analyzed using GraphPad Prism 7.

For manuscripts utilizing custom algorithms or software that are central to the research but not yet described in published literature, software must be made available to editors and reviewers. We strongly encourage code deposition in a community repository (e.g. GitHub). See the Nature Portfolio guidelines for submitting code & software for further information.

Data

Policy information about availability of data

All manuscripts must include a data availability statement. This statement should provide the following information, where applicable:

- Accession codes, unique identifiers, or web links for publicly available datasets
- A description of any restrictions on data availability
- For clinical datasets or third party data, please ensure that the statement adheres to our policy

ScRNA-Seq data have been deposited to Gene Expression Omnibus (GEO) with access number GSE143997

Research involving human participants, their data, or biological material

Policy information about studies with human participants or human data. See also policy information about sex, gender (identity/presentation), and sexual orientation and race, ethnicity and racism.

Reporting on sex and gender	This study did not involve human research participants.
Reporting on race, ethnicity, or other socially relevant groupings	This study did not involve human research participants.
Population characteristics	This study did not involve human research participants.
Recruitment	This study did not involve human research participants.
Ethics oversight	This study did not involve human research participants.

Note that full information on the approval of the study protocol must also be provided in the manuscript.

Field-specific reporting

Please select the one below that is the best fit for your research. If you are not sure, read the appropriate sections before making your selection.

☒ Life sciences ☐ Behavioural & social sciences ☐ Ecological, evolutionary & environmental sciences

For a reference copy of the document with all sections, see nature.com/documents/nr-reporting-summary-flat.pdf

Life sciences study design

All studies must disclose on these points even when the disclosure is negative.

Sample size	The SAN primordium appears at E10.5 based on histological structure and becomes further mature and fully functional at E13.5. Shox2 homozygous mice died at mid-gestation due to cardiac defects, while Shox2Cre/Cre;Nkx2-5F/F mice (dKO) survived to postnatal day 0 (P0). Embryos or embryonic hearts were collected at different embryonic stages (E10.5, E11.5, E12.5, E13.5, E14.5, P0).
Data exclusions	No data were excluded from the analyses.
Replication	All experiments were repeated at least three times unless specifically indicated.
Randomization	We used the littermate wild-type mice as controls and transgenic mice as the experimental group.
Blinding	At 6-8 weeks of age, transgenic mice were mated, then timed pregnant mice were euthanized to collect embryos and embryonic hearts. And PCR was performed on embryos tail genomic DNA to further identify embryos genotype.

Reporting for specific materials, systems and methods

We require information from authors about some types of materials, experimental systems and methods used in many studies. Here, indicate whether each material, system or method listed is relevant to your study. If you are not sure if a list item applies to your research, read the appropriate section before selecting a response.

Materials & experimental systems

n/a	Involved in the study	
☐	☒	Antibodies
☒	☐	Eukaryotic cell lines
☒	☐	Palaeontology and archaeology
☐	☒	Animals and other organisms
☒	☐	Clinical data
☒	☐	Dual use research of concern
☒	☐	Plants

Methods

n/a	Involved in the study	
☒	☐	ChIP-seq
☒	☐	Flow cytometry
☒	☐	MRI-based neuroimaging

Antibodies

Antibodies used	anti-Shox2 (homemade; 1:500), anti-Nkx2-5 (sc-8697, Santa Cruz; 1:500), anti-Tbx3 (sc-17871, Santa Cruz; 1:500), anti-Hcn4 (ab32675, Abcam; 1:500), anti-Cx40 (sc-20466, Santa Cruz; 1:250), anti-GFP (sc-9996, Santa Cruz; 1:500). The secondary antibodies

	were all purchased from Life Technologies and used at 1:1000 dilution: donkey-anti rat (A21208, A21209), donkey-anti rabbit (A31573), donkey-anti mouse (A21202, A31571), donkey-anti goat (A32849, A11058, A11055).
Validation	Shox2 is a rabbit polyclonal antibody, recommended for detection of Shox2 of mouse and human origin by Western Blotting (1:100), immunofluorescence (1:500); Nkx2-5 is a goat polyclonal antibody, recommended for detection of Nkx2-5 of mouse, and human origin by Western Blotting (1:1000), immunofluorescence (1:500); Tbx3 is a goat polyclonal antibody, recommended for detection of Tbx3 of mouse, and human origin by Western Blotting (1:1000), immunofluorescence (1:500); Hcn4 is a rat monoclonal antibody, recommended for detection of Hcn4 of rat, mouse, and human origin by Western Blotting (1:5000), immunofluorescence (1:500), IHC-P (1:10 - 1:100), Flow Cyt (1:50); Cx40 is a goat polyclonal antibody, recommended for detection of Cx40 of mouse, and human origin by Western Blotting (1:1000), immunofluorescence (1:500); GFP is a mouse monoclonal antibody, recommended for detection of GFP and GFP mutant fusion proteins by Western Blotting (starting dilution 1:200, dilution range 1:100-1:1000), immunoprecipitation [1-2 µg per 100-500 µg of total protein (1 ml of cell lysate)], immunofluorescence (starting dilution 1:50, dilution range 1:50-1:500), flow cytometry (1 µg per 1 x 106 cells) and solid phase ELISA (starting dilution 1:30, dilution range 1:30-1:3000).

Animals and other research organisms

Policy information about studies involving animals; ARRIVE guidelines recommended for reporting animal research, and Sex and Gender in Research

Laboratory animals	All transgenic mice are at 6-8 weeks of age, C57BL/6 background. The mice species are Shox2Cre, Shox2HA-Neo, Nkx2.5F/F, and R26RmTmG.
Wild animals	The study did not involve the wild animals.
Reporting on sex	This information has not been collected.
Field-collected samples	The study did not involve samples collected from the field.
Ethics oversight	The animal experiments in this study were approved by the Institutional Animal Care and Use Committee of Tulane University and by the Animal Ethic Committee of Fujian Normal University.

Note that full information on the approval of the study protocol must also be provided in the manuscript.
